# The Gerontologist

**DOI:** 10.1093/geroni/igaf122.1235

**Published:** 2025-12-31

**Authors:** Joseph Gaugler

**Affiliations:** University of Minnesota, Minneapolis, Minnesota, United States

## Abstract

The Gerontologist®, first published in 1961, features applied, multidisciplinary research and analysis of social issues related to human aging. Our journal informs the broad community of disciplines and professions involved in understanding the aging process and providing care to older people. Contributions are welcome from scholars from many fields, including social and psychological sciences, social work, health sciences, the humanities and arts, education, public health, technology, political science, and public policy. The Gerontologist® prioritizes submissions that feature, refine, or create new conceptual/theoretical models that advance our understanding of aging. The Gerontologist® encourages manuscript submissions of various types: research articles; review articles; measurement articles; forums; and those commissioned by the Editors including book and media reviews, International Spotlights, and award-winning lectures. The Gerontologist®’s robust, interdisciplinary articles and issues are augmented by a series of timely special issues and collections on key topics of relevance to gerontological scholarship.

